# Spatial Patterns of Natural Protected Areas and Construction of Protected Area Groups in Guangdong Province

**DOI:** 10.3390/ijerph192214874

**Published:** 2022-11-11

**Authors:** Yi Deng, Ziyi Mao, Jinling Huang, Faling Yan, Shenghai Han, Anqi Li

**Affiliations:** 1 School of Architecture and Urban Planning, Guangzhou University, Guangzhou 510006, China; 2 School of Resources and Planning, Guangzhou Xinhua University, Guangzhou 510310, China; 3 Guangzhou CAOMUFAN Ecological Research Co., Ltd., Guangzhou 510000, China

**Keywords:** natural protected areas, spatial pattern, kernel density analysis, groups of natural protected areas

## Abstract

The fragmentation of protected areas is a common issue in global conservation, which means a new approach to planning and management needs to be explored. In this paper, we proposed the concept of a group of natural protected areas (GNPA) and studied the construction of GNPAs. Firstly, the spatial distribution characteristics of 1363 natural protected areas (NPAs) in Guangdong Province were qualitatively studied. The overall spatial pattern among NPAs and the spatial distribution characteristics of mountain ranges, river basins, urbanization level and economic density were analyzed, and the relationship between the distribution of NPAs and physical geography and social development was clarified. Then, the geographical concentration index, nearest index and Gini coefficient were used for quantitative analysis. The geographical concentration index was 24.6, and the nearest neighbor index was 0.8. The Gini coefficients of the spatial distribution of NPAs in Guangdong Province were Gini = 0.956 and C = 0.044. These indices proved that the overall spatial patterns of NPAs in Guangdong Province had the tendency and characteristics of agglomeration. On this basis, 29 agglomeration areas were constructed using kernel density analysis and the natural break point classification method. According to the requirements of spatial connectivity and management feasibility, combined with the characteristics of physical geography, ecosystems and biodiversity, 32 GNPAs were constructed based on the reasonable adjustment of 29 agglomeration areas. Using Geodetector statistics to analyze the spatial stratified heterogeneity of the GNPAs, the results showed that mountain range, water system, population density, economic density and urbanization level were all factors that could explain the clustering distribution of the natural protected areas. The most important factor was mountain range (*p* = 0.190), followed by population density (*p* = 0.162). The 32 GNPAs covered the most representative natural ecosystems in the province and had compact spatial organization, a close ecological relationship and feasible unified management, which means they could aid in resolving the fragmentation of protected areas and improving management efficiency.

## 1. Introduction

### 1.1. Research Background

Natural protected areas (NPAs) are essential in the preservation of global biodiversity and in the maintenance of the ecological security of all countries [1,2,3]. In recent years, due to the constraints of multi-dimensional conflicts such as economy and population and the impact of rapid urbanization development, the fragmentation of NPAs has become increasingly severe and has become a common problem in the field of global nature protection. Therefore, new approaches in planning and management need to be explored [4,5,6,7,8].

In Guangdong Province, the construction of NPAs is very important, and this province was the first to construct nature reserves in China. According to survey data regarding NPAs, in 2019 there were 1363 NPAs in Guangdong Province after integration and optimization, meaning the province ranked first in China. However, the area of individual NPAs is small and scattered, and the pattern of NPAs in the whole province is fragmented. The NPAs’ low conservation effect, high conservation cost and low management efficiency seriously affect their conservation efficiency. Therefore, an NPA system must urgently be established in Guangdong Province, with national parks as the main body. This will enable the spatial distribution rule of NPAs in Guangdong Province to be studied. Furthermore, groups of natural protected areas (GNPAs) can be identified and created, the ecological relationship between NPAs within groups and their components can be strengthened, and a protection and management mode for NPAs can be created [9,10].

### 1.2. Related Work

The cognition and measurement of fragmentation is the basis for improving the conservation efficiency of NPAs. Fragmentation refers to the process by which a large continuous distribution of natural habitat is separated into many smaller patches by other unsuitable habitats under the influence of human activities and natural disturbances [11,12]. The multiple negative impacts of the fragmentation process, such as the reduction of natural habitat area, the reduction of connectivity between habitat patches, and the continuous decline of habitat quality, will pose serious challenges to human well-being and regional sustainable development [13,14,15]. Initially, a density-dependent reproduction and survival simulation model was developed, and it was concluded that the degree of fragmentation of nature reserves was positively correlated with the species extinction probability [16]. The four main factors leading to the fragmentation of forest reserves are the effect of the non-random sampling of the original forest, reduced forest size, isolation and edge effects [17]. The measurement methods used in landscape fragmentation are also constantly being optimized, and the introduction of the degree of landscape division, splitting index and effective mesh size has further improved the measurement of landscape fragmentation [18]. Due to improvements in research regarding fragmentation, habitat fragmentation is now defined as a landscape-scale process, which promotes research regarding the ecological impact of habitat fragmentation [19]. Recent studies have shown that only 9.7% of the Earth’s conservation network can be defined as structurally connected [20]. As a result, more attention has been paid to habitat fragmentation worldwide, and the concept of “low-impact areas” has emerged to calculate the global bio-community fragmentation rate. Nature reserves are the main method of resisting habitat fragmentation, and they are a cornerstone of biodiversity conservation [21]. China’s NPAs have fuzzy boundaries, cross ranges and have NPA division, which aggravate the fragmentation of natural ecosystems. However, there has been no mention of any measures that might be used to optimize NPAs [22,23]. In order to reduce the fragmentation of NPAs, many scholars have discussed the integration and optimization of NPAs, and because different types of NPAs serve different dominant ecosystems, various integration methods for use in NPAs based on different standards have been proposed [24,25]. However, NPAs are still fragmented after optimization and integration. After analyzing change trends with a degree of landscape fragmentation and spatial heterogeneity and complexity, scholars introduced the theory of landscape ecology. By using GIS technology to analyze the characteristics of the landscape fragmentation of NPAs’ interior and exterior and its driving factors, it was found that compared with natural factors, human factors had a more significant effect on fragmentation [25,26,27].

Studies regarding fragmentation reveal the importance of the connectivity of NPAs to sustain ecological processes [28]. Therefore, the questions of how to restore the fragmentation of NPAs, enhance the ecological connectivity of NPAs and improve conservation efficiency have become important topics for scholars worldwide [29,30,31,32]. Graph theory analysis was applied to the loss and fragmentation of NPAs and refined elements of landscape connectivity [33]. The restoration of landscape connectivity requires the establishment of reliable and efficient connectivity models and assessment indicators [34,35]. The perfection of graphic theory allows for the precise quantitative assessment of landscape connectivity [34]. Simultaneously, specific strategies such as the establishment of ecological corridors, the connection and formation of ecological networks, the maintenance of biodiversity and the optimization of the NPA system have been gradually improved [36,37,38,39].

Exploring the spatial distribution characteristics of NPAs and making overall planning for NPAs from a macro perspective will contribute to the connectivity of NPAs [40]. At present, there have been some research results on the spatial distribution of NPAs. The research on the spatial distribution characteristics of nature reserves has been carried out from many aspects. Wang Shu Yi et al. analyzed the spatial distribution of nature reserves in southwest China from the perspective of an isochronal circle model [41]; Cao Yue et al. used the wilderness mapping method to identify the wilderness in mainland China [42]; Jiang Chao et al. and Ma Tong Hui et al. analyzed the spatial distribution characteristics of nature reserves using different aspects of an aggregation degree correlation index [43,44]. The present research preliminarily proves that there exists unbalance and certain aggregation in the spatial distribution of NPAs [41,42,43,44,45]. To further quantify the spatial distribution characteristics of suitable habitats and important ecosystems, Geodetector was used to measure spatial stratified heterogeneity [46,47]. In order to improve the conservation effect, it is suggested to combine and associate NPAs, supplement vacancies and appropriately adjust the boundaries of NPAs to enhance NPAs’ aggregation [48,49,50].

On the basis of the abovementioned research, the concept of a protected area network and GNPAs based on spatial aggregation arose. Lin Peng et al. first introduced the concept of a network of nature reserves and proposed construction methods, emphasizing the necessity of the construction of a nature reserve network [51]. Jingxiao proposed the concept of nature reserve groups earlier but only discussed optimization methods and approaches toward its pattern and function [52]. Many scholars have carried out studies regarding the ecological benefit, benefit measurement and sustainable development of nature reserve groups in the Qinling Mountains [53,54,55]. The concepts, data and methods related to nature reserve groups have continued to be enriched. Hong Liu et al. commented on the concept of nature reserve groups, but it was only mentioned as part of the scope of their research, and an in-depth study was not carried out [56]. With the gradual implementation of national parks, Fan Jie et al. used national parks as their main research object and conducted a regional function and feasibility study regarding national park groups [57]. However, because nature reserves and national parks are only part of the system of nature reserves, these studies regarding a single system do not reflect the characteristics of ecosystem integrity, and they are difficult to implement. On the basis of analyzing the current situation in the Wuyi Mountain nature reserve, Yang Rui et al. further proposed the concept of a Wuyi Mountain GNPA based on the positioning of national parks [58]. In summary, the study of GNPAs has expanded from GNPAs being a single category of protected areas to a system of protected areas with national parks as the main body [59]. The research and development in this field tends to be systematic, which lays the foundation to further the construction and improvement of the system and mechanism.

There are complex ecological and sociological considerations in defining the areas of NPAs. It is difficult to simply expand the area of an NPA. Fragmentation could be reversed through spatial connectivity and management linkages. The abovementioned studies show that spatial agglomeration is a prerequisite for establishing ecological corridors, improving connectivity and establishing unified management among NPAs. The qualitative and quantitative analysis of the spatial distribution of NPAs aids our understanding of the spatial distribution characteristics and relationships of NPAs under the constraints of ecological and social environment, and it can help to identify the potential for improving spatial connectivity through factors such as spatial aggregation, the integrity of physical geographic units and management feasibility.

### 1.3. Research Aim and Framework

Based on the abovementioned research, in this paper we propose the concept of a “protected natural area group” (GNPA). A GNPA is an ecological unit in a compact area and a close ecological connection composed of at least three protected natural areas with more than one high-level or large-scale protected natural area with important conservation value as the core in an important ecological functions area. GNPAs could feasibly establish closer ecological links, and on this basis, unified and collaborative management can be established to effectively improve the overall conservation effect of natural ecology.

Landscape pattern generally refers to its spatial pattern, which refers to the spatial arrangement and combination of landscape elements with different sizes and shapes, including the type, number, spatial distribution and configuration of landscape component units. Different types of patches can show random, uniform or aggregated distribution in space [60]. It is the concrete embodiment of landscape heterogeneity and the result of various ecological processes at different scales [61]. Landscape pattern can be quantitatively described by landscape index. It usually includes patch shape index, patch density, landscape richness index, contagion index, landscape connectivity index and so on. Since the goal of this study is to build a GNPA with spatial connectivity potential and the possibility of effective management, the geographical concentration index, nearest proximity index and Gini index were selected to quantitatively analyze the distribution characteristics of GNPA. In addition, in order to quantify the spatial stratified heterogeneity of GNPA, this study used Geodetector for further analysis to provide data support on the theoretical basis of optimizing the GNPAs, so as to make the division of GNPAs more scientific.

**Figure 1:**
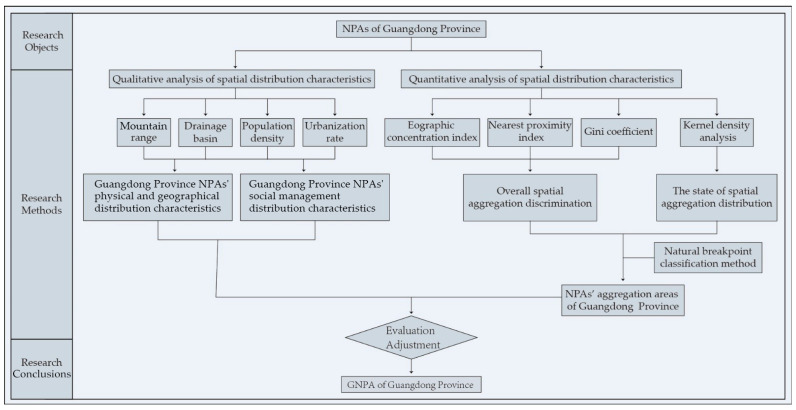
Research framework.

The overall concept of this study was as follows. In view of the current distribution fragmentation of natural protected areas in Guangdong Province, the spatial pattern of NPAs was taken as the research object, and the relationship between the distribution of NPAs and physical geographical units and social development was clarified through qualitative analysis. The geographical concentration index, nearest proximity index and Gini coefficient were used to identify the overall agglomeration of the NPAs’ spatial distribution. On this basis, kernel density analysis and the natural breakpoint classification method were used to construct the agglomeration area of NPAs. Then, through the analysis of the unity and management feasibility of the natural geographic units, ecosystems and protected objects in each agglomeration area, the units and scopes of GNPAs in Guangdong Province were constructed after reasonable adjustment (Figure 1). These can provide a research basis for further improvement of the efficiency of nature conservation and the fragmentation pattern of natural protected areas through group management.

## 2. Data Sources and Methods

### 2.1. Data Sources

In this study, sealed data of the National Forestry and Grassland Administration from December 2020 was used. The research object was integrated and optimized NPAs in Guangdong Province.

Basic geographic information data was obtained from the National Catalogue Service for Geographic Information; this database includes river systems, residential areas and facilities, transportation, place names and notations, etc. The population and economic development data were taken from the economic and social development bulletins of the People’s Government at all levels.

Land cover data of Guangdong Province was taken from the global 30-m land cover fine classification product from 2020 released by the Aerospace Information Institute, Chinese Academy of Sciences.

Remote sensing image data was obtained from the existing remote sensing image data platform.

Natural resource data was mainly provided by the Forestry Administration of Guangdong Province, supplemented by government open data queries.

The data of NPAs was mainly based on the data archived in the survey of NPAs in Guangdong Province in 2019, supplemented by other data collections and literature downloads.

### 2.2. Methods

In this study, we qualitatively analyzed the spatial distribution characteristics of NPAs in Guangdong Province, including mountain ranges, drainage basins and social development factors. At the same time, the geographic concentration index, nearest proximity index and Gini coefficient were used to quantitatively analyze the spatial distribution trends and characteristics of NPAs. The aggregation and distribution areas of NPAs in Guangdong Province were determined through kernel density analysis. In addition, the Geodetector model was used to further quantify the spatial stratified heterogeneity of the distribution of GNPAs. The analytical methods and main indices are shown in Table 1 and Table 2.

## 3. Results

In this study, we qualitatively analyzed the correlation between geographical unit distribution characteristics and the spatial distribution of NPAs in Guangdong Province. Combined with the quantitative calculation of the spatial distribution characteristics of NPAs, the results are presented in the following section.

### 3.1. Spatial Distribution Characteristics of NPAs in Guangdong Province

The relationship between the distribution of NPAs and the spatial distribution characteristics of mountain ranges, river basins, population density, economic density and urbanization level in Guangdong Province was analyzed.

#### 3.1.1. Mountain-Related Spatial Distribution Characteristics

Most of the mountains in Guangdong Province follow the trend of geological structures, with the majority following the north-east to south-west trend. The central mountain ranges are the Lotus Mountains, Luofu Mountains, Julian Mountains, Qingyun Mountains and Yunwu Mountains. After integration, it was shown that about 536 NPAs are distributed in the central mountains and mountainous areas, covering an area of about 1.8658 million hectares, accounting for 63.02% of the total area of Guangdong Province.

**Figure 2:**
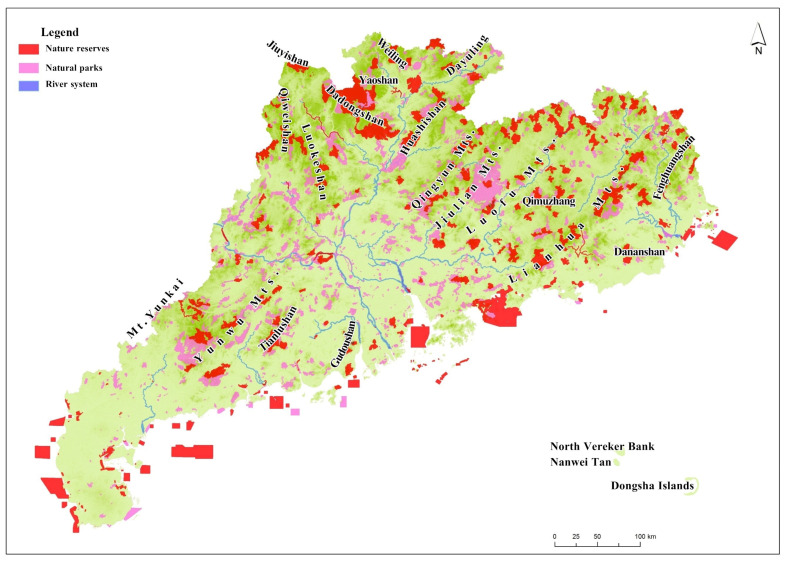
Spatial location relationship between NPAs and mountain ranges.

Most of the nature reserves in Guangdong Province are distributed in mountainous areas, except for marine nature reserves, and the distribution trend coincides with the mountain trend. Meanwhile, the mountain peaks are more densely distributed in the mountain range, and the more NPAs that are distributed, the larger the area proportion is (Figure 2).

#### 3.1.2. River-Related Spatial Distribution Characteristics

There are many rivers in Guangdong Province, mainly divided into the Zhujiang River Basin, the Hanjiang River Basin and the Guangdong, Guangxi and Hainan coastal River Basins. The principal rivers are the Dongjiang River, Beijiang River, Xijiang River and the Zhujiang Delta Rivers.

In terms of river basins, it was shown that about 484 NPAs are distributed within 10 km of the main rivers in Guangdong Province, covering an area of about 592,100 hectares, accounting for 20% of the total NPAs in Guangdong Province.

In terms of river basins, excluding 106 coastal marine nature reserves, 979 NPAs are in the six major river basins, accounting for 81.43% of the total NPAs in Guangdong Province.

**Figure 3:**
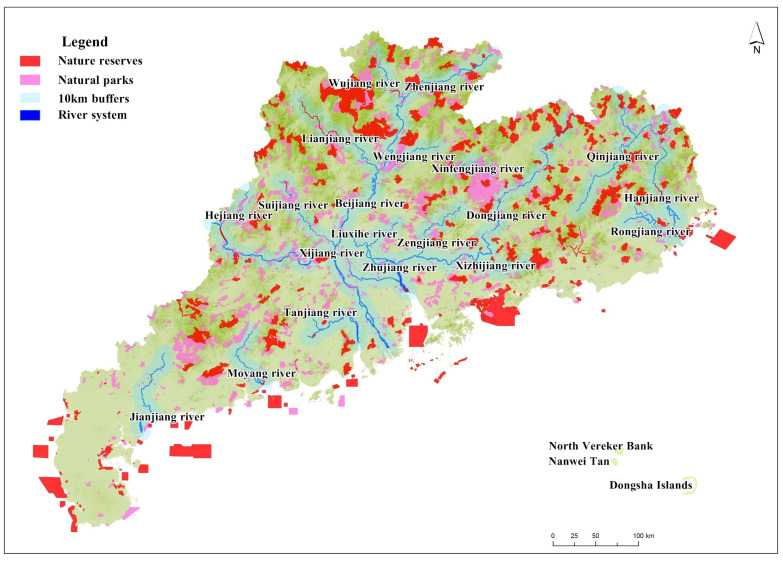
Spatial location relationship between NPAs and river basins.

The spatial distribution trend of the river basins in Guangdong Province was shown to be similar to that of the mountain ranges. The distribution trend of the nature reserves is coincident with that of the river basins. There are many NPAs in the same eco-geographical unit (Figure 3).

#### 3.1.3. Population-Density-Related Spatial Distribution Characteristics

**Figure 4:**
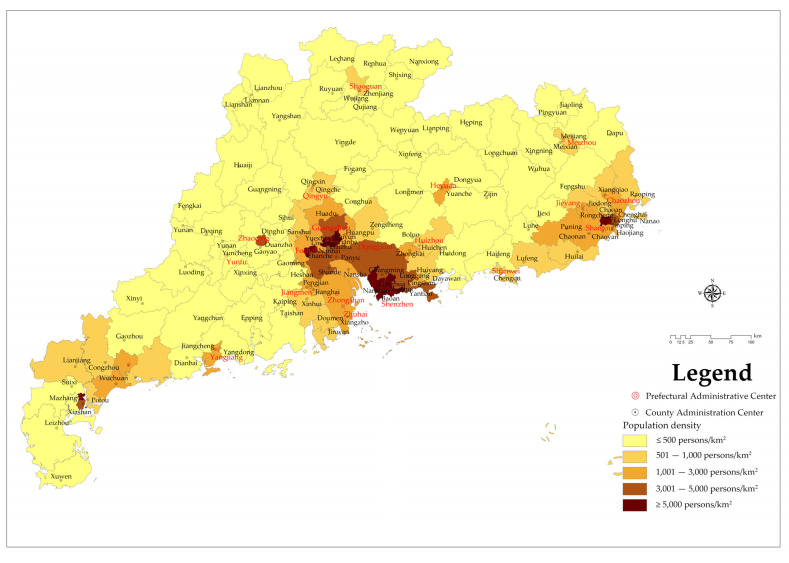
Hierarchical distribution of population density.

There are 145 counties and districts in Guangdong Province, which are divided into five grades according to the population density of each county as follows, Grade I: ≤500 people/km^2^; Grade II: 501–1000 people/km^2^; III: 1001–3000/km^2^; IV: 3001–5000/km^2^; V: >5000–km^2^. The population density grades of the counties and districts in Guangdong Province are shown in Figure 4.

According to the analysis data, there are 711 NPAs with 22,795 million hectares in Grade I areas, accounting for 65.7% of the total number of nature reserves in Guangdong Province. In areas with a population density of Grade V, there is the lowest number of nature reserves, with 26 nature reserves with an area of 17,400 hectares, accounting for 2.41% of the total natural reserves in Guangdong Province.

As can be seen from Figure 3, the population of Guangdong Province is mainly concentrated in coastal areas such as the Zhujiang River Delta region, with no NPAs in the areas with the highest population densities and a relatively high proportion of NPAs in the areas with the lowest population densities. The higher the level of population density, the fewer NPAs are distributed in the region; therefore, the distribution of NPAs in Guangdong Province is inversely proportional to its population density.

#### 3.1.4. Economic-Density-Related Spatial Distribution Characteristics

**Figure 5:**
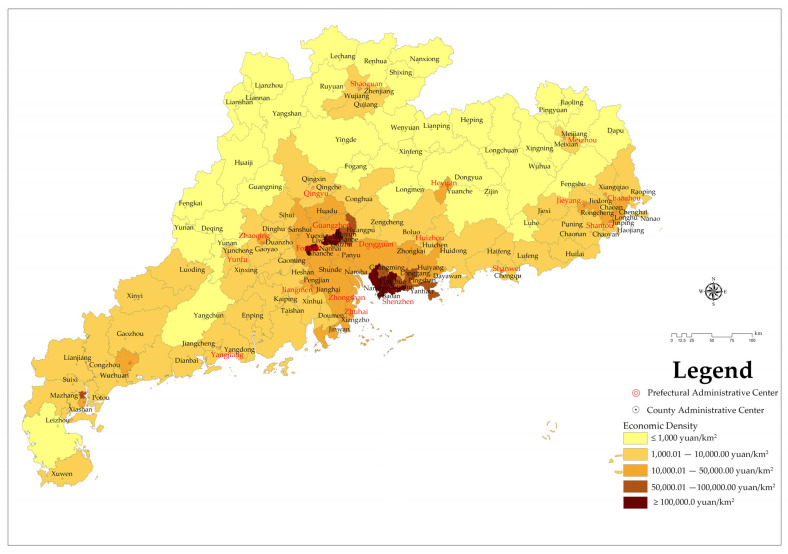
Hierarchical distribution of economic density.

Based on the 2019 GDP of each county in Guangdong Province and each county’s land area, each county’s economic density was calculated and divided into five grades. Among them, Grade I: ≤1000/km^2^; Grade II: 1000.01~10,000/km^2^; Grade III: 10,000.01~50,000/km^2^; Grade IV: 50,000.01~100,000/km^2^; Grade V: >100,000/km^2^. The economic density grades of the counties and districts in Guangdong Province are shown in Figure 5.

According to the analysis data, NPAs are most extensively distributed in the Grade I economic density areas in Guangdong Province, with 435 NPAs covering an area of 1.5362 million hectares, accounting for 40.42% of the total number of NPAs in Guangdong Province. In the Grade V economic density area in Guangdong Province, there are 23 NPAs covering a total area of 13,500 hectares, accounting for 2.13% of the total nature reserves in Guangdong Province.

The above analysis shows that the distribution of economic density is consistent with the distribution of population density, and there is a positive correlation between them. Therefore, the higher the economic density, the less extensive the distribution of NPAs. The distribution of NPAs in Guangdong Province is inversely proportional to its economic density.

#### 3.1.5. Urbanization-Level-Related Spatial Distribution Characteristics

**Figure 6:**
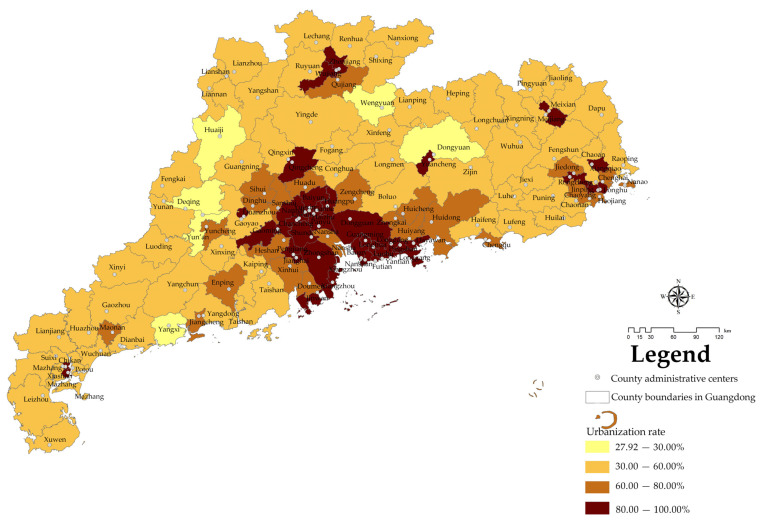
Hierarchical distribution of urbanization rate.

Based on the urban population and total population of each city and county in Guangdong Province in 2019, the urbanization rate of each county was calculated. The urbanization rate of each city and county in Guangdong province was divided into four grades according to the classification method used for the urbanization level: Grade I: ≤30%; Grade II: 30–60%; Grade III: 60–80%; Grade IV: 80–100%. The range of urbanization rates by county is shown in Figure 6.

According to the analysis data, the number of NPAs in counties with Grade I urbanization rates in Guangdong Province is fewest; there are 73 NPAs with 271,800 hectares in total, accounting for 6.65% of the total NPAs in Guangdong Province. At the same time, there are 631 NPAs with an area of 1970.8 million hectares in Grade II counties, accounting for 57.47% of the total NPAs in Guangdong Province.

According to these results, the overall urbanization rate of the counties in Guangdong Province is at the middle level, and a low urbanization rate in counties is rare. Most of the nature reserves are distributed in the regions with low urbanization rates, meaning the urbanization rate of each county in Guangdong Province is consistent with the population density and economic density.

Based on the analysis of the abovementioned social development factors, it can be concluded that the more prosperous the regional development is, the lower the distribution of NPAs in the region, and the distribution of NPAs in Guangdong Province is negatively correlated with the overall development level of the region.

### 3.2. NPAs’ Aggregation in Guangdong Province

#### 3.2.1. Geographic Concentration Index Analysis

The geographic concentration index is an important index to measure the concentration degree of NPAs in Guangdong Province. The value of G is within the range of 0–100; a lower value indicates a lower concentration and a more balanced distribution. The higher the value, the higher the concentration, and the uneven distribution.

According to the calculation formula of the geographic concentration index, the number of NPAs in different cities and the proportion of NPAs in the whole province were considered. The geographical concentration index G = 24.6 was calculated.

In the whole of Guangdong Province, NPAs have ideal average distribution in 21 administrative areas. The NPAs’ spatial distribution had a geographic concentration index G′ = 21.8, and the actual calculation results G > G′ show that the geographic concentration index of NPAs in Guangdong Province is higher than the ideal value and shows an aggregated spatial distribution.

#### 3.2.2. Nearest Proximity Index Analysis

The nearest proximity index represents the average measurement of the distance between the centroid of each factor and the centroid position of its nearest neighbors. When R = 1, it indicates that the distribution type of protected land is random. When R > 1, the distribution tends to be uniform. When R < 1, it tends to be an agglomeration distribution.

**Figure 7:**
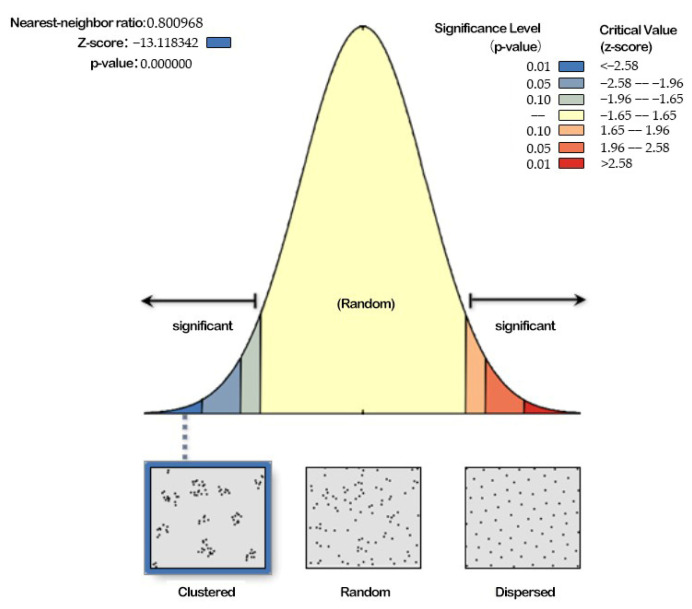
Average nearest neighbor analysis results of NPAs in Guangdong Province.

According to the average nearest neighbor analysis results for the NPAs in Guangdong Province, the nearest neighbor index is 0.8; that is, the average distance is less than the average distance in the assumed random distribution (the expected average distance) (Figure 7). Therefore, the NPAs in Guangdong Province are clustering elements and present an aggregation distribution.

#### 3.2.3. Gini Coefficient Analysis

The Gini coefficient is mainly used to reflect the distribution of spatial elements in discrete regions. Theoretically, the Gini index ranges from 0 to 1. The larger the Gini coefficient, the higher the concentration degree in the region, and the smaller the Gini coefficient, the higher the equilibrium degree.

By substituting the data of NPAs in Guangdong Province into the Gini coefficient calculation formula (N = 21), the Gini coefficient of the spatial distribution of protected areas in Guangdong province can be obtained as Gini = 0.956, C = 0.044. The Gini value of the NPAs in Guangdong Province is relatively high, which means that the NPAs in Guangdong Province are distributed centrally in each administrative region and are unevenly distributed in space.

#### 3.2.4. Spatial Kernel Density Analysis

The analysis results from the geographical concentration index, nearest neighbor index and Gini coefficient showed that although the individual NPAs in Guangdong Province are small and dispersed, their spatial distribution is not balanced, and the overall spatial distribution is still clustered. This proves that spatial clustering is not a local phenomenon and the idea of establishing GNPAs is feasible in the whole province. However, these three indices could not be used to describe the specific agglomeration area of the NPAs.

**Figure 8:**
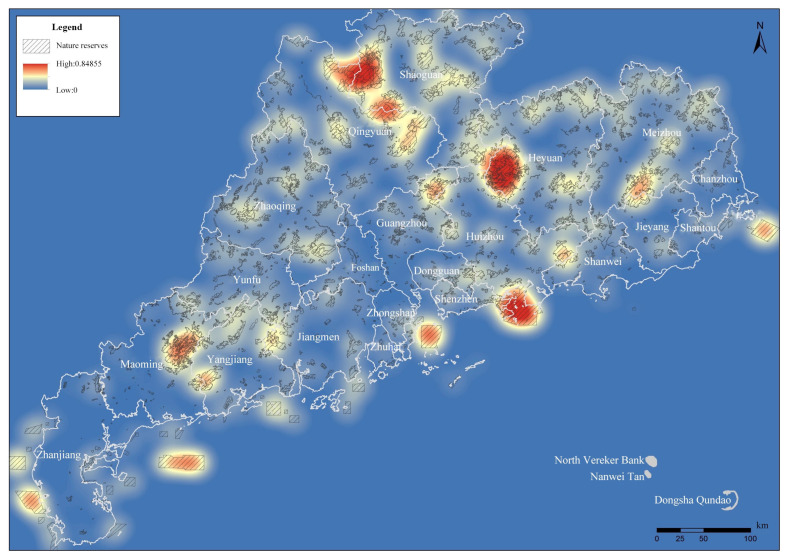
The results of integrated optimization of NPAs and kernel density analysis superposition.

In order to further clarify the agglomeration area based on the kernel density research method, in this study we conducted kernel density spatial clustering analysis using the province’s integrated and optimized NPA data. Under the 25,000 m bandwidth setting condition, the kernel density surface obtained was relatively consistent with the actual situation (Figure 8), and the density value was between 0 and 0.85. The analysis results show areas with high degrees of aggregation in the whole province.

### 3.3. Construction of GNPA

This analysis proved that the NPAs in Guangdong Province have spatial aggregation and have the potential to improve spatial connectivity and reverse the fragmentation pattern. By refining the spatial aggregation area of NPAs, and by considering the unity of natural geographic units, ecosystems and protected objects in each aggregation area and the feasibility of management, we could reasonably select NPAs with close spatial proximity and close ecosystem connection to construct GNPAs.

#### 3.3.1. Identification of Spatial Agglomeration Area

Based on these ideas, the spatial kernel density analysis results are the most important bases for the determination of the distribution of GNPAs. According to the aggregation area ranges of NPAs based on the results of kernel density analysis, combined with the distribution of NPAs and the characteristics of the mountain and basin systems, optimization and adjustment were carried out. Finally, the spatial distribution of a GNPA in Guangdong Province was obtained.

At the same time, in order to facilitate the analysis of the distribution of NPAs in various density ranges, the results of the kernel density analysis of NPAs in the province were divided into 10 levels using the natural breakpoint classification method. The higher the level, the higher the distribution density of the NPAs in its neighborhood space.

**Figure 9:**
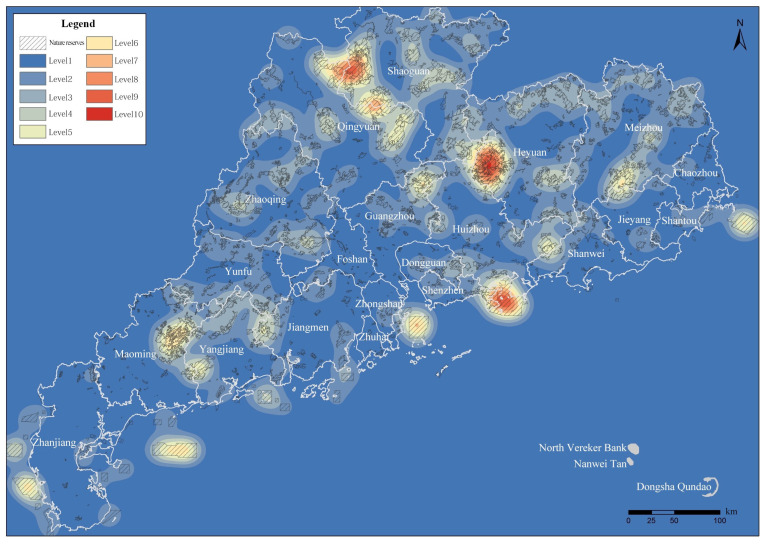
NPAs’ kernel density classification map.

In the areas with density grades 1 and 2, the spatial aggregation degree of NPAs is lower, and the distribution is scattered, which does not meet the basic conditions of the agglomeration area. In the kernel density areas above grade 3, the total number of NPAs accounts for 34.47% of the total number of NPAs in the province, and the total area of NPAs accounts for 71.47% of the total area of NPAs in the province. In kernel density areas above grade 4, the total number of NPAs accounts for 12.35% of the total number of NPAs in the province, and the total area of NPAs accounts for 43.45% of the total area of NPAs in the province. Finally, the areas with kernel densities of grade 5 or above account for 4.15% of the number and 22.47% of the area of the total NPAs in the province (Figure 9).

**Figure 10:**
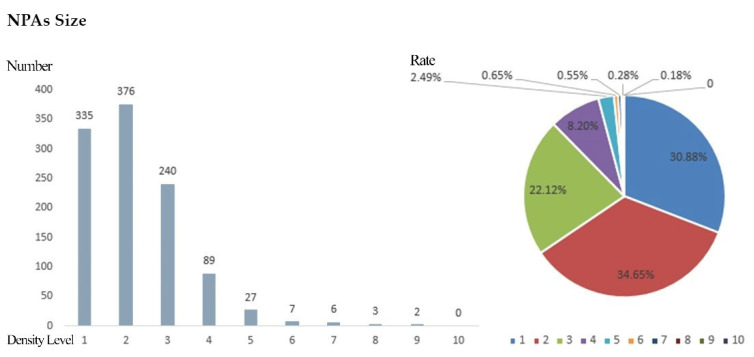
Statistics regarding the size of NPAs at different levels.

**Figure 11:**
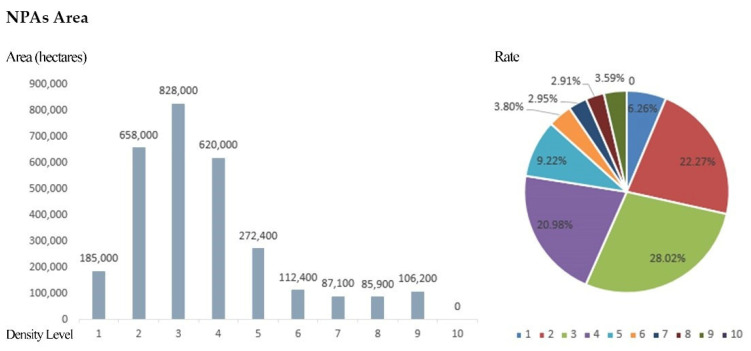
Statistics regarding the area of NPAs at different levels.

The statistics from areas with every level of kernel density affected the area and quantity of NPAs (Table 3, Figure 10 and Figure 11), according to the results from the calculation and analysis of the spatial distribution, quantity and area proportion of NPAs in each kernel grade area. This was based on the preliminary determination of the scope of the NPAs’ relatively concentrated area within the province (agglomeration zone) as a preliminary basis to delimit the GNPA.

**Figure 12:**
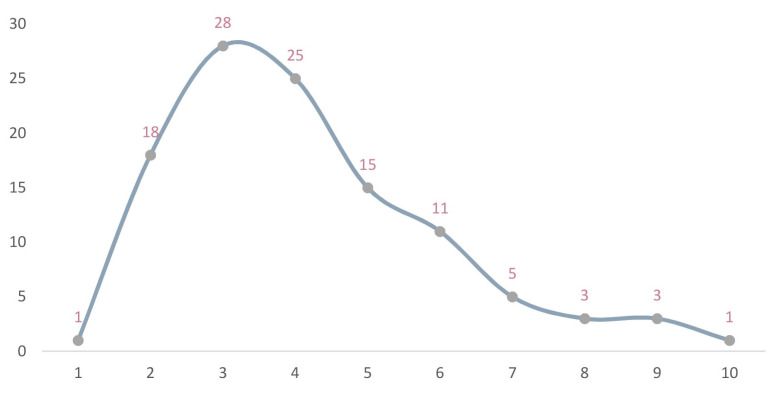
Trend chart of changes in agglomeration area quantity.

**Figure 13:**
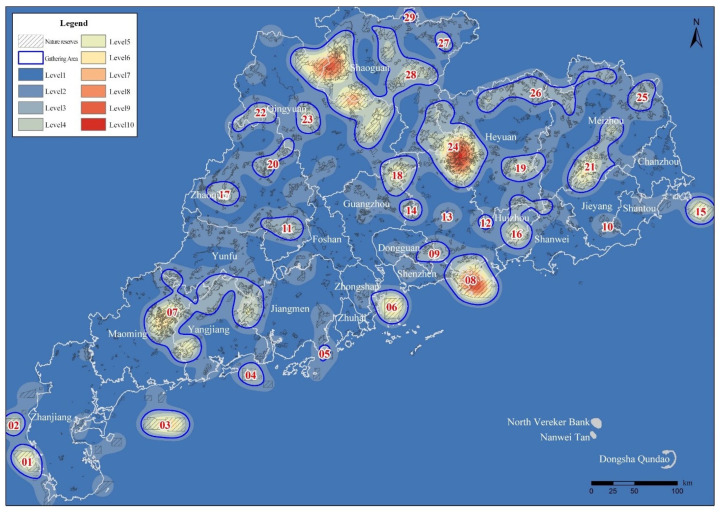
Agglomeration area distribution of NPAs in Guangdong Province. (The numbers in the figure are the sequential numbers of NPAs Agglomeration areas).

When the initial kernel density level was 3, the maximum number of agglomeration areas was 28 (Table 4 and Figure 12). In order to comprehensively study the scope, NPAs were included. To satisfy the basic spatial aggregation degree, the combination of NPAs of every type and level of distribution in all concentration areas was included. The study team ultimately determined a density of grade 3 or more in areas designated as NPA agglomeration areas on the basis of the following analysis. According to the spatial distribution continuity of areas with kernel density grades of 3 or above, 29 NPA agglomeration areas were divided (Figure 13).

#### 3.3.2. Optimization and Adjustment Based on Ecological Space Elements

**Figure 14:**
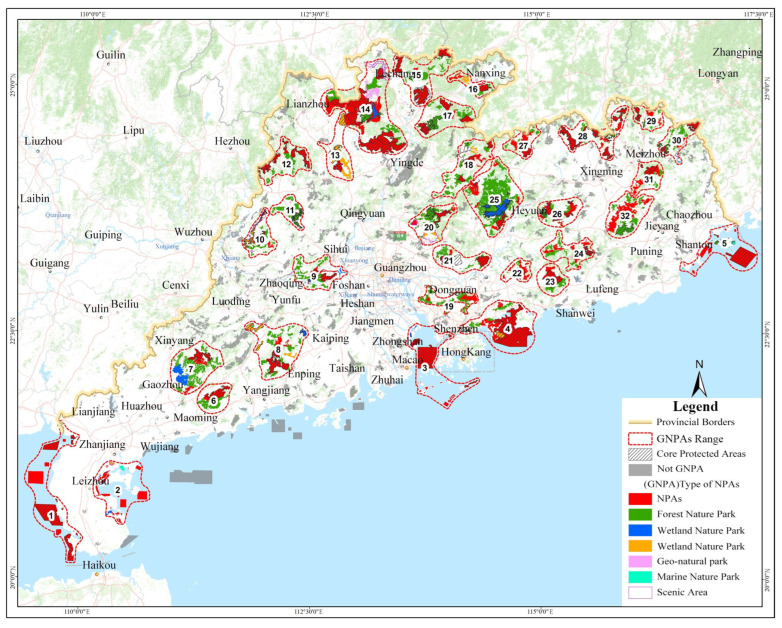
Distribution of GNPAs in Guangdong Province. (The numbers in the figure are the sequential numbers of GNPAs).

Based on the distribution of natural reserve agglomeration areas, the differences in physical geographic units, main ecosystems and the biodiversity of each agglomeration area were optimized and adjusted. The agglomeration areas with close ecological process connections were expanded or merged. The agglomeration areas belonging to different vegetation zones or vegetation zones with large spatial spans that are not conducive to cluster management were reduced or divided. Natural protected areas involving multiple areas were optimized and adjusted into different agglomeration areas. Agglomeration areas with a small number of protected natural areas or no core protected natural areas were excluded. The detailed analysis and construction processes are shown in Table 5, and the result of the construction of natural protected areas in the province is shown in Figure 14.

#### 3.3.3. Construction Results

After this analysis and conclusions, a total of 32 GNPAs with a total area of 2.032 million hectares were constructed in the province, accounting for 68.77% of the total NPAs in the province, including 409 NPAs, accounting for 37.70% of the total number of NPAs in the province. There are 99 core NPAs with a total area of 1.0328 million hectares, accounting for 83.98% of the total area of core NPAs and 50.83% of the total area of GNPAs (Figure 14).

## 4. Conclusions 

### 4.1. Measures of Spatial Stratified Heterogeneity and Attribution of GNPAs

GNPAs in Guangdong Province are established on the premise that the GNPAs are distributed in a specific space and have a certain degree of aggregation relationship. To understand which natural or social factors contribute to this clustering distribution pattern, it is necessary to measure and analyze the spatial stratified heterogeneity of GNPAs.

The Geodetector method is a statistical method that quantitatively describes spatial stratified heterogeneity and reveals the driving factors behind it [62], including tools such as Factor detector and Interaction detector. It is usually used to calculate the importance of the influencing factor and the influence of the interaction of the factor [63,64]. In this study, the Factor detector in the Geodetector was used to measure the spatial stratified heterogeneity of the dependent variable, and the degree of explanation of a single influencing factor to the spatial stratified heterogeneity of the GNPAs was clarified, which was expressed by the statistic q. At the same time, Interaction detector is used to measure the degree of interaction between the two influencing factors and clarify the degree of interaction between the influencing factors.

**Figure 15:**
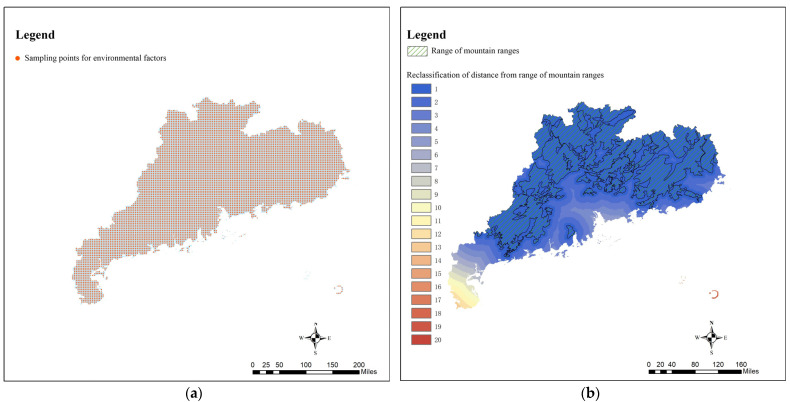

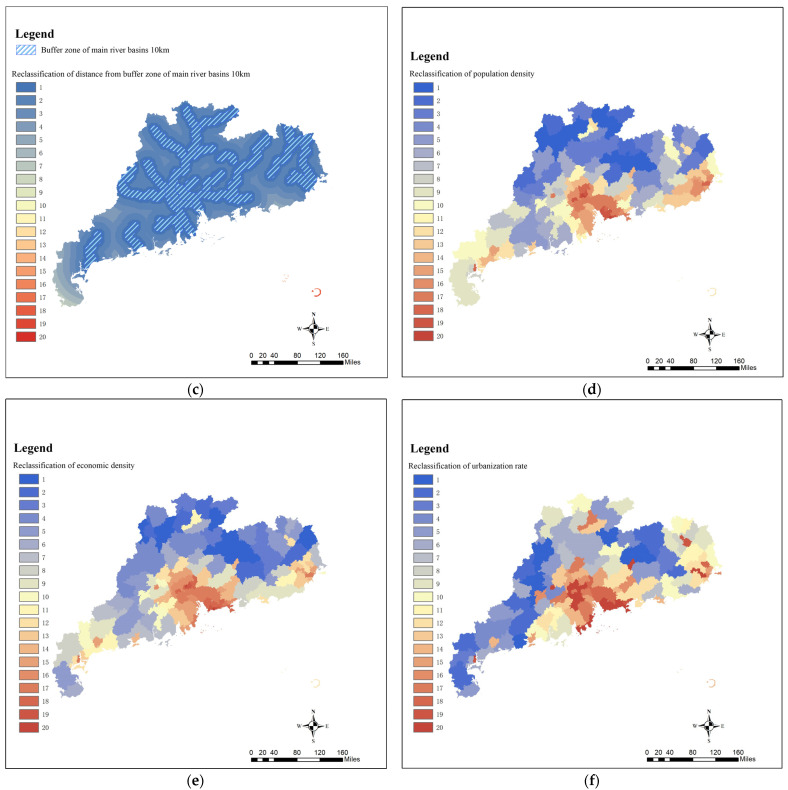
(**a**) 5 km × 5 km sampling points for environmental factors; (**b**) Reclassification of distance from range of mountain ranges; (**c**) Reclassification of distance from buffer zone of main water systems 10 km; (**d**) Reclassification of population density; (**e**) Reclassification of economic density; (**f**) Reclassification of urbanization rate.

In this study, a 5 km × 5 km fishnet sampling point (Figure 15a) was used for sampling, and the distribution of mountains (Figure 15b), distribution of river basins (Figure 15c), population density (Figure 15d), economic density (Figure 15e) and urbanization rate (Figure 15f) in Guangdong Province were taken as influencing factors X; the result of the GNPAs’ kernel density analysis (Figure 9) is Y. The above data are used for spatial stratified heterogeneity analysis.

**Figure 16:**
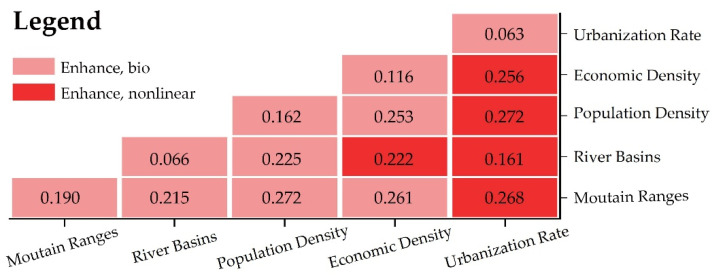
The results of the Interaction detector.

The attribution and formation mechanism analysis of the spatially stratified heterogeneity of GNPAs is as follows:

(1) The Fa ctor detector results showed that the q statistic of the mountain range distribution was the largest among all factors, indicating that mountain range distribution was the main influencing factor affecting the distribution of the GNPAs. Other influencing factors were population density (q = 0.162), economic density (q = 0.116), river distribution (q = 0.066) and urbanization (q = 0.063). The *p* values of the five influencing factors are all 0.00, indicating that the significance of their influence on the GNPAs is extremely high (Table 6). Therefore, the spatial cluster distribution of GNPAs in Guangdong Province is the result of the joint action of natural ecological process and social development process. Mountains’ range distribution is the main influencing factor. This is because the type of GNPA in Guangdong Province is mainly forest ecosystem which is given priority, while mountains in natural geographical units have a relatively good natural environment and a variety of habitats, so the correlation between the distribution of GNPAs and the distribution of mountains is much higher than for other natural factors, such as the distribution of river basins.
(2) Among all the social influencing factors, the *p* value of population density is the largest, indicating that population density is the most important social factor affecting the distribution of GNPAs. This is because the site selection of GNPAs in Guangdong Province tends to avoid areas with large human interference, which can be defined from different aspects by population density, economic density or urbanization rate. In terms of data sources, population density includes town-level data at the city level, which is more refined in spatial classification than the other two types of data, so it can more truly and accurately reflect the intensity of land development and utilization and the intensity of human disturbance. Therefore, in this study, population density can better reflect the influence of social factors on the spatial distribution of GNPAs than the other two types of data.
(3) Interaction detection is mainly used to explain whether the two influence factors act independently or have interactive effects, and the results are as follows (Figure 16). Mountain distribution ∩ urbanization rate, river distribution ∩ urbanization rate, river distribution ∩ economic density, population density ∩ urbanization rate and economic density ∩ urban rate are “Enhancement, nonlinear”, indicating that the explanatory power of the interaction of the above five groups of two influencing factors is much greater than that of single factors. The interaction between the other factors is “Enhancement, bio”, that is, after the interaction of the two factors, the explanatory power is greatly enhanced compared with the original single-factor explanatory power. In this study the effect of the selected factors in the interaction detection did not appear as “Weaken, nonlinear”, “Weaken, uni-” and “Independent” in the Interaction detection. The interaction of influencing factors was greater than that of single factors on the spatial distribution of GNPA, that is, the interaction of each influencing factor positively strengthened the influence of each factor. Therefore, the spatial distribution of GNPAs was the result of the comprehensive action of multiple influencing factors.
(4) Therefore, under the influence of ecological factors and social factors, the spatial distribution of GNPAs shows the following characteristics: they are closely related to the spatial coupling of mountain systems and river basins; close coupling with the spatial distribution of main protected objects; mutually exclusive with the existing urban construction and development space. Finally, 28 relative aggregation NPAs were formed in the physical geographic units of the Lianhua Mountain Range, Lufu Mountain Range, Jiulian Mountain Range, Qingyun Mountain Range and Yunwu Mountain range, and the major river systems of the Pearl River Basin, Han River Basin and Yue-Gui-Qiong coastal river basin, showing a group distribution

The spatial distribution characteristics of the clustered NPAs provide the possibility for its integrated management. Without expanding the area of NPAs, integrated management of GNPAs and strengthening the connection between NPAs will help to solve the fragmentation trend of NPAs.

### 4.2. GNPAs Based on Spatial Aggregation of NPAs and Its Ecological Significance

Based on the results of kernel density analysis and the natural breakpoint method, 28 agglomerations were divided, and 32 GNPAs were constructed by optimizing the agglomerations according to their spatial distribution characteristics, accounting for 68.77% of the total natural protected areas in the province. These 32 GNPAs, covering all precious resources, are all key areas that are rich in biodiversity, accounting for 83.98% of the total area of the core natural protected areas. The protected areas within these 32 GNPAs have the characteristics of spatial aggregation and connectivity potential, and their ecosystem- and biodiversity-related characteristics are relatively consistent, which provides an integrated direction for the construction of ecological corridors to prioritize construction within groups and strengthen construction among key groups. These 32 GNPAs cover seven different types and management systems of NPAs, including nature reserves, forest parks, wetland parks, geological parks, desert parks, marine parks and scenic spots. This is also conducive to the improvement of the construction project and management systems of NPAs through intra-group cooperation. Therefore, the construction of GNPAs will become a new method of improving the conservation effect, reducing the cost of conservation and improving the efficiency of management and conservation.

In conclusion, the construction of GNPAs is very important to resist the fragmentation of the protected area system in Guangdong Province, and it provides a theoretical basis for further studies regarding the landscape security pattern and ecological characteristics of GNPAs. Further research should be carried out based on the spatial division of GNPAs to explore the establishment of a more perfect conservation management system and mechanism in accordance with the characteristics of its ecosystem. At the same time, we will explore the establishment of trial management methods for GNPAs, carry out special pilot projects, strengthen inter-regional and inter-administrative cooperation and promote the implementation of GNPAs.

## Figures and Tables

**Table 1:** Analytical methods and main indices.

Research Methods	Analysis Purposes	Formulas	Symbolic Meaning	Research Significance	Remark
Geographic Concentration Index	To measure the concentration of research subjects.	$G=100\times \sqrt{\sum_{i=1}^{n}{\left(\frac{x_{i}}{m}\right)}^{2}}$	“G” is the geographic concentration index, “xi” is the number of objects in the “i” urban area, “n” is the number of regions and “M” is the total number of objects.	It reflects the spatial distribution concentration of protected areas in Guangdong Province.	To make the kernel density analysis results more reasonable, the spot-like distribution of NPAs and the influence of the area size of NPAs on the kernel density analysis results were considered. In this study, the province was divided into 1 km × 1 km grids using fishing-net-tools based on the kilometer grid distribution data of NPAs in the province. The grid area was 1 square kilometer, that is, 100 hectares. The data of integrated NPAs were intersected with the grid central point data, and the grid central point falling in the integrated NPAs was taken as the object of spatial kernel density analysis.
Nearest Proximity Index	The average nearest neighbor tool measures the distance of a position between each element centroid and its nearest element centroid. Then, the average of all of those nearest neighbor distances is computed.	$R=\frac{\bar{r_{1}}}{\bar{r_{E}}}=2\sqrt{D}$	“r1” is the actual nearest distance, “rE” is the theoretical nearest distance, “D” is the point density. When “R = 1”, this indicates that the NPAs’ distribution type is random. When “R > 1”, the distribution tends to be uniform. When “R < 1”, it tends to be concentrated.	To judge the spatial distribution types of protected areas in Guangdong Province.	
Gini Coefficient	It is mainly used to reflect the distribution of spatial elements in discrete regions.	$G_{ini}=\frac{-\sum p_{i}Inp_{i}}{InN}$ C=1−Gini	“Gini” is the Gini coefficient value, “C” is the distribution equilibrium degree, “N” is the number of zones and “Pi” is the proportion of the number of NPAs in the “i” area in Guangdong Province. The higher the Gini coefficient, the lower the distribution equilibrium degree.	The equilibrium degree of the spatial distribution of geographical phenomena in protected areas in Guangdong Province was analyzed.	
Kernel Density Analysis	Density analysis is a process of interpolation through discrete point or line data. The points falling into the search area are analyzed with different weights, and the change in the local density of events is measured.	$f\left(x\right)=\frac{1}{nh}\sum_{i=1}^{n}k\left(\frac{x-x_{i}}{h}\right)$	The distance between data point “xi” and “x” is used to determine the effect of “xi” on estimating the density of point “x”. H > 0 is a smoothing parameter, called bandwidth, which is an artificially selected threshold. In actual research, the bandwidth should be adjusted according to the specific situation to obtain a kernel density surface that is more consistent with the actual situation.	To determine the aggregation and distribution areas of protected natural areas in Guangdong Province.	
Factor_ detector	Indicates the extent to which each independent variable explains the spatial differentiation of the dependent variable.	q = 1 − $\frac{\sum_{h=1}^{L}N_{h}\sigma_{h}^{2}}{N\sigma^{2}}$	$\mathrm{Where}\;h=1\ldots ,\;L\;\mathrm{is}\;\mathrm{the}\;\mathrm{stratification}\;\mathrm{of}\;\mathrm{variable}\;Y\;\mathrm{or}\;\mathrm{factor}\;X;\;N_{h}$$\mathrm{and}\;N\;\mathrm{are}\;\mathrm{the}\;\mathrm{number}\;\mathrm{of}\;\mathrm{units}\;\mathrm{in}\;\mathrm{stratification}\;h\;\mathrm{and}\;\mathrm{the}\;\mathrm{whole}\;\mathrm{area}\;\mathrm{respectively};\;\sigma_{h}^{2}$$\mathrm{and}\;\sigma^{2}$ are the variances of the layer h and the whole region Y values, respectively.	Indicates the extent to which each independent variable explains the spatial differentiation of the dependent variable in Guangdong Province.	

**Table 2:** Kernel density grading standards.

Kernel Density Level	Kernel Density Value Range	Kernel Density Level	Kernel Density Value Range
1	d ≤ 0.09	6	0.56 < d ≤ 0.66
2	0.19 < d ≤ 0.28	7	0.66 < d ≤ 0.75
3	0.28 < d ≤ 0.38	8	0.75 < d ≤ 0.80
4	0.38 < d ≤ 0.47	9	0.80 < d ≤ 0.85
5	0.47 < d ≤ 0.56	10	0.85 < d

**Table 3:** Statistics regarding the number and area of NPAs at different levels.

Density Level	Reserve Size		Nature Reserve Area	
	Number	Rate (%)	Area (Hectares)	Rate (%)
1	335	30.88	185,000	6.26
2	376	34.65	658,000	22.27
3	240	22.12	828,000	28.02
4	89	8.2	620,000	20.98
5	27	2.49	272,400	9.22
6	7	0.65	112,400	3.8
7	6	0.55	87,100	2.95
8	3	0.28	85,900	2.91
9	2	0.18	106,200	3.59
10	0	0	0	0
A combined	1085	100	295.5	100

**Table 4:** Statistics regarding the number of agglomeration areas of different initial kernel density grades.

Initial Kernel Density Level	Agglomerations Number	Initial Kernel Density Level	Agglomerations Number
1	1	6	11
2	18	7	5
3	28	8	3
4	25	9	3
5	15	10	1

**Table 5:** Construction processes of GNPAs.

Category	Agglomeration Area Number	Protected Area Group Number	Name of the Protected Area Group	Construction Analysis of GNPAs
Basic type (Take agglomeration as the basic scope)	11	9	Xijiang–Dinghu Mountain	This kind of agglomeration area has a high degree of agglomeration, good connectivity, similar or identical natural geographical characteristics and the feasibility of unified and collaborative management. The main ecosystem and habitats are the same, and the main protection object is the same or similar. GNPAs can be established on the basis of agglomeration areas.
	12	22	Lianghua	
	18	20	Liuxi River–Nankun Mountain	
	19	26	Kanghe–Qimuzhang	
	22	12	Dachouding group	
Gather type (Partially adjusted basic on agglomeration)	06	3	Zhujiangkou	This kind of agglomeration area has the same internal physical geographical characteristics and single topography. The integrity of protected areas is high, but the number is small. The surrounding area is adjacent to more fragmented protected areas. Some protected areas around the agglomeration areas with the same main ecosystem, the same habitat and the same or similar main protection objects were included to form GNPAs.
	08	4	Daya Bay	
	09	19	Yinpingzui–Daling Mountain	
	15	5	HanJiangKou	
	17	10	Black stone top–Yanyan	
	20	11	Suijing–Sea of Bamboo	
	23	13	Yang Mountain–Yingxi	
	25	30	Yinna Mountain–Fengxi	
	27	16	Guanyindong–Green Ridge Mountain	
Merge type (Combining several agglomeration areas as the basic scope)	01/02	1	Eastern shore of the Gulf Tonkin	The protected areas in this kind of agglomeration area are small and few in number. Adjacent agglomeration areas are close to each other, with the same main ecosystem, the same habitat and the same or similar main protected objects. Therefore, several agglomeration areas are merged to form GNPAs.
	14/13	21	Luofu Mountain–Elephant head Mountain	
Split type (The large agglomeration area is divided into protected area groups)	07	6	Ehuangzhang	This kind of agglomeration area covers a large area, and there are many protected areas in the area. The core protected areas are far away from each other, and the terrain is different or in different mountain ranges and river basins. Therefore, considering the management feasibility, the unity of habitats and the main protected objects, the agglomeration area was divided into several GNPAs with the main protected area as the core and surrounding fragmented sites.
		7	Yunwu Mountain	
		8	Tiannu Mountain	
	16	23	Lianhua Mountain	
		24	WuqinZhang–Wudun	
	21	31	Tongguzhang	
		32	Dabei Mountain–Baxiang Mountain	
	24	18	Qingyun Mountain–Huangniushi	
		25	Wanlvhu	
	26	27	Jiulian Mountain	
		28	Maple Dam–Huangtian	
		29	Changtan–Zhen Mountain	
	28, 29	14	Nanling–Shimental	
		15	Danxia Mountain	
		17	Che Right Ridges–Small pits	
Supplementary type	_	2	Lake Light Rock–Techen Island	This group has a lower kernel density, but the number of covered protected sites is large, and the protection level is high. Therefore, it is supplemented as a new GNPA.
The excluded area	03	-	-	The results of the kernel density analysis of such an agglomeration area are affected by several protected areas around the area. Although the results of kernel density analysis are high, the coverage of protected areas is lower and the grade is low, which does not meet the basic requirements for the establishment of protected areas. Therefore, GNPAs are not set up in such areas.
	04			
	05			
	10			

**Table 6:** The results of Factor detector.

	q Statistic	*p* Value
Mountain Range	0.190	0.000
River Basins	0.066	0.000
Population Density	0.162	0.000
Economic Density	0.116	0.000
Urbanization Rate	0.063	0.000

## Data Availability

Data presented in this study are available on request from the corresponding authors. The data was not released due to requirements of the local government’s data confidentiality policy.
